# Two new *Mallinella* species from southern China (Araneae, Zodariidae)

**DOI:** 10.3897/zookeys.296.4622

**Published:** 2013-04-30

**Authors:** Chi Jin, Feng Zhang

**Affiliations:** 1 College of Life Sciences, Hebei University, Baoding, Hebei 071002, P. R. China

**Keywords:** Spider, *Mallinella*, taxonomy, new species, China

## Abstract

Two new species of the spider genus *Mallinella* Strand, 1906 are reported from China: *Mallinella sphaerica* **sp. n.** (male, female) from Tianmu Mountain, Zhejiang Province and *Mallinella pluma* **sp. n.** (male) from Daming Mountain, Guangxi Zhuang Autonomous Region.

## Introduction

The spider family Zodariidae is represented by 78 genera and 1068 known species mainly from Africa, Australia and Asia (Platnick 2013), but only 36 species in 7 genera (*Asceua* Thorell, *Cydrela* Thorell, *Mallinella* Strand, *Storenomorpha* Simon, *Heradion* Dankittipakul & Jocqué, *Zodariellum* Andreeva & Tyschchenko and *Heliconilla* Pakawin et al.) have been reported from China (Platnick 2013; Zhang et al. 2012a, b; Dankittipakul et al. 2012).

The genus *Mallinella* was established by Strand in 1906, was redefined by Jocqué (1991) and differs from the other zodariids by the following characters: the sternum with triangular extensions fitting in coxal concavities, the presence of a row of short spines in front of the tracheal spiracle, and a well-developed conductor which is a terminal excrescence of the tegulum (Jocqué, 1991). Until now, 202 *Mallinella* species have been reported worldwide (Platnick 2013), including 17 *Mallinella* species from southern China (Song and Kim 1997; Song et al. 1999; Wang et al. 1999; Yin and Yan 2001; Bao and Yin 2002; Zhang and Zhu 2009; Wang et al. 2009; Wang et al. 2009; Zhang et al. 2011, Zhang et al. 2012a; Dankittipakul et al. 2012).

While examining spiders collected from Tianmu Mountain, Zhejiang Province, and Daming Mountain, Guangxi Zhuang Autonomous Region of southern China, we found two species new to science – *Mallinella sphaerica* sp. n. and *Mallinella pluma* sp. n. that are described herein.

## Material and methods

All specimens were kept in 75% ethanol, examined and measured under a Tech XTL-II stereomicroscope. Drawings of *Mallinella sphaerica* sp. n. were prepared under a Nikon SMZ 1500 stereomicroscope equipped with a drawing tube, and *Mallinella pluma* sp. n. under a Leica M165C stereomicroscope equipped with a drawing tube. The photos were taken with a Leica M205A stereomicroscope equipped with a DFC450 CCD. Carapace length was measured from the anterior margin to the centre of the posterior margin. Eye size was measured as the maximum diameter of the lens in dorsal or frontal view. The leg measurements are presented as total length (femur, patella, tibia, metatarsus, tarsus). The epigyne was cleaned in a warm solution of potassium hydroxide (KOH) and transferred to 75% ethanol for drawing. All measurements are in millimeter. All specimens studied are deposited in the Museum of Hebei University (MHBU), Baoding, China.

## Abbreviations

**ALE** anterior lateral eyes

**AME** anterior median eyes

**C** conductor

**CD** copulatory ducts

**E** embolus

**EP** epigynal plate

**MA** median apophysis

**MOA** median ocular area

**PLE** posterior lateral eyes

**PME** posterior median eyes

**RTA** retrolateral tibial apophysis

**S** spermathecae

**T** tegulum.

## Taxonomy

### Zodariidae Thorell, 1881

#### Mallinella (Mallinella) sphaerica sp. n.

urn:lsid:zoobank.org:act:6591D5D0-F5D3-4498-8C74-3900F1A8270C

http://species-id.net/wiki/Mallinella_sphaerica

Figs 1 –15

##### Type material.

Holotype ♂, CHINA, *Zhejiang Province*: Tianmu Mountain (30°18'N, 119°27'E), alt. 262m, Lin’an City, 25 July 2011, C. Jin leg. Paratype: 1♀, same data as holotype.

##### Diagnosis.

According to Dankittipakul et al. 2012, this species should belong to sub-group 3 of the *fronto* species-group. Males can be distinguished from these in other *Mallinella* species by the spherical median part of the median apophysis and the irregular rolled apex of the conductor in ventral view (Figs 10, 14). The female is extremely similar to that of *Mallinella cymbiforma* Wang, Yin & Peng, 2009, but differs from the latter by the thinner copulatory ducts (Figs 8, 13), the wider posterior margin of the epigynal plate (Figs 7, 12), and MOA wider at the back (MOA wider at the front in *Mallinella cymbiforma*).

##### Etymology.

The specific name is a Latin adjective and refers to the spherical median part of the median apophysis.

##### Description.

Male (holotype). Total length 6.27; prosoma 3.11 long, 2.14 wide; opisthosoma 2.96 long, 1.99 wide. Diameters of eyes: AME 0.20, ALE 0.15, PME 0.15, PLE 0.15. Distances between eyes: AME–AME 0.08, AME–ALE 0.10, ALE–ALE 0.55, PME–PME 0.13, PME–PLE 0.25, PLE–PLE 0.78, ALE–PLE 0.05. MOA 0.48 long, anterior 0.50, posterior 0.43. Clypeal height 0.75. Labium 0.58 long, 0.50 wide. Sternum 1.35 long, 1.28 wide. Leg measurements: leg I 10.36 (2.55, 0.77, 2.35, 2.65, 2.04), II 9.90 (2.50, 0.92, 2.04, 2.60, 1.84), III 9.29 (2.35, 0.82, 1.94, 2.65, 1.53), IV 12.09 (2.70, 0.92, 2.60, 3.88, 1.99). Leg formula: 4123.

**Figures 1–6.**
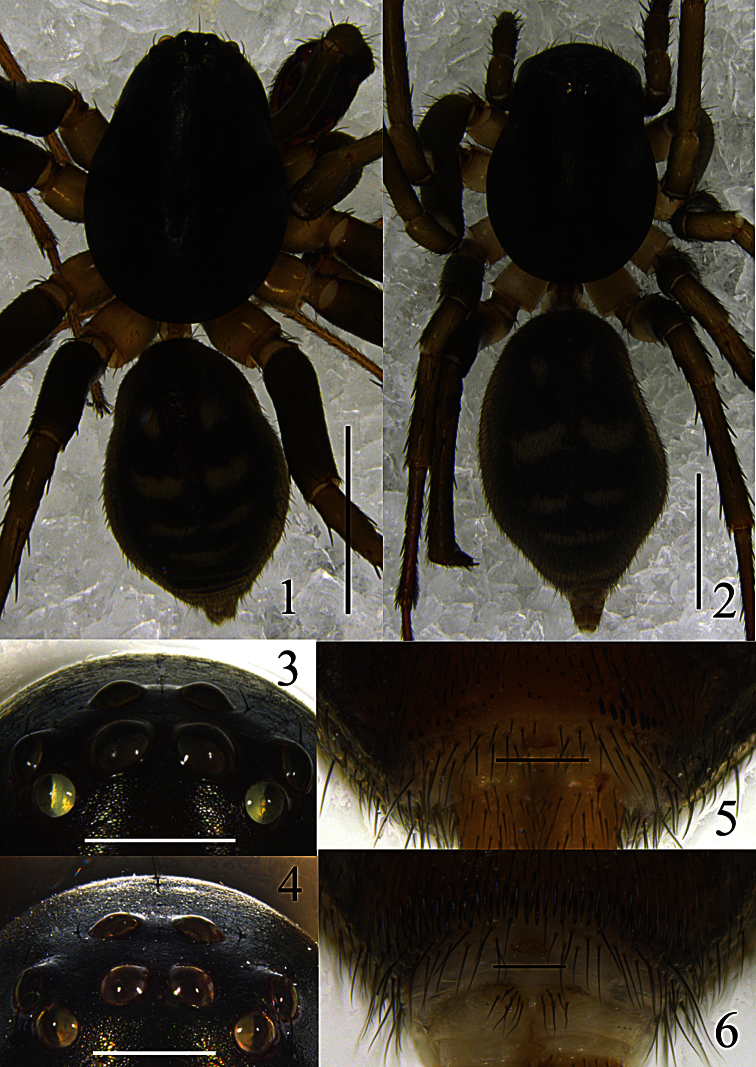
*Mallinella sphaerica* sp. n., **1** male habitus, dorsal view **2** female habitus, dorsal view **3** male ocular area, frontal view **4** female, ocular area, frontal view **5** male, posterior ventral spines, ventral view **6** female, posterior ventral spines, ventral view. Scale bars: 2 mm (**1–2**); 0.5 mm (**3–4**); 0.2 mm (**5–6**).

Carapace (Fig. 1) blackish-brown; fovea black, slightly swollen. Both eye rows (Fig. 3) procurved in dorsal view. Clypeus dark brown. Chelicerae dark brown, distally yellowish-brown. Endites yellowish-brown. Labium triangular, dark brown, distally yellowish-brown. Sternum brown and furnished with sparse black setae, lateral margin with small, pointed extensions fitting in coxal concavities. Legs brown, femora dark brown, coxae yellowish-brown; metatarsi II–IV distally with ventral hair tufts. Opisthosoma (Fig. 1) oval, longer than wide, dorsum with three pairs of white lateral patches (the third connected), followed by one transverse white patch centrally. Dorsal scutum reddish-brown, about half as long as opisthosoma. Venter grey-black, covered with three irregular longitudinal white stripes; posterior ventral spines thin (Fig. 5), arranged in a single row. Spinnerets pale yellow.

Palpal organ (Figs 9–11, 14, 15). RTA digitiform, slightly wider at base, gradually tapering towards pointed apex. Cymbial fold broad, approximately half as long as cymbium. Apex of median apophysis bifid, with sharply pointed tips on both ends; the median part of median apophysis spherical; baso-retrolateral fold narrow, triangular, apex pointed. Apex of conductor irregularly rolled. Embolus bifurcate, lateral ramus shorter than mesal ramus, with pointed apices.

Female (paratype). Total length 8.26; prosoma 3.57 long, 2.55 wide; opisthosoma 4.28 long, 2.75 wide. Diameters of eyes: AME 0.20, ALE 0.18, PME 0.18, PLE 0.18. Distances between eyes: AME–AME 0.08, AME–ALE 0.10, ALE–ALE 0.60, PME–PME 0.15, PME–PLE 0.28, PLE–PLE 0.88, ALE–PLE 0.05. MOA 0.53 long, anterior width 0.45, posterior width 0.50. Clypeal height 1.03, Labium 0.63 long, 0.63 wide. Sternum 1.53 long, 1.53 wide. Leg measurements: leg I 8.68 (2.45, 0.97, 1.94, 1.79, 1.53), II 8.47 (2.19, 0.97, 1.79, 1.99, 1.53), III 8.00 (2.19, 0.92, 1.68, 2.14, 1.07), IV 10.34 (2.14, 1.02, 2.24, 3.06, 1.68). Leg formula: 4123.

Coloration and pattern (Fig. 2) as in male, but the color of coxae slightly paler than in male. Median furrow and scutum indistinct.

**Figures 7–11.**
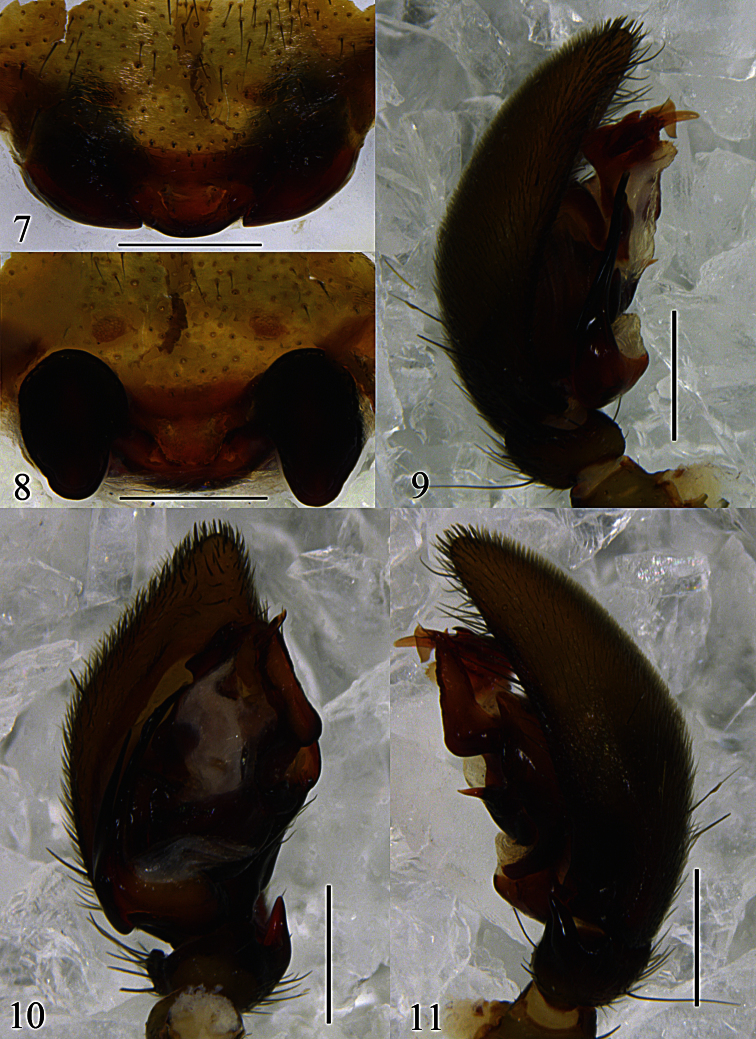
*Mallinella sphaerica* sp. n., **7** epigyne, ventral view **8** vulva **9** left male palp, prolateral view **10** same, ventral view **11** same, retrolateral view. Scale bars: 0.5 mm (**7–11**).

**Figures 12–15.**
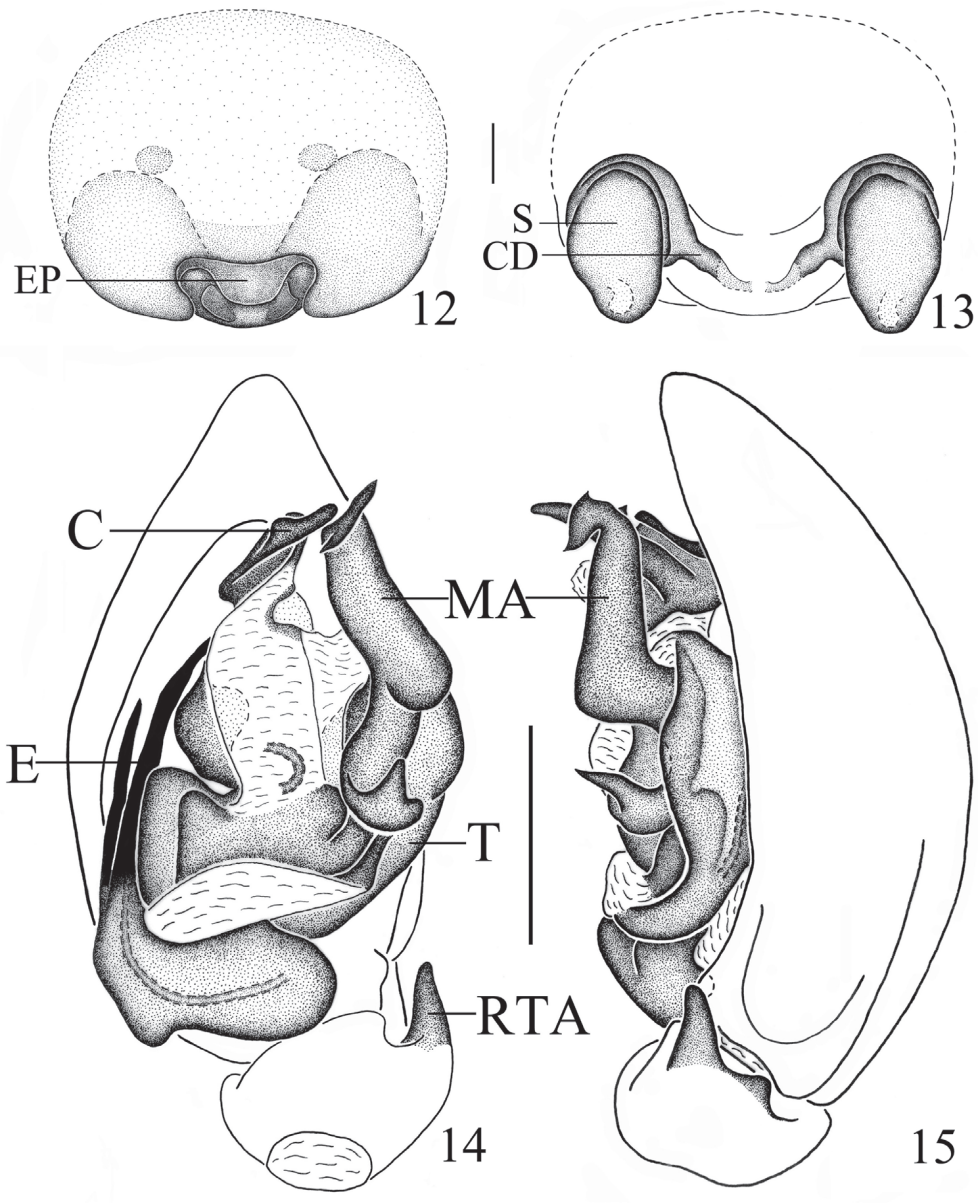
*Mallinella sphaerica* sp. n., **12** epigyne, ventral view **13** vulva **14** left male palp, ventral view **15** same, retrolateral view. Scale bars: 0.2 mm (**12–13**); 0.5 mm (**14–15**).

Epigyne-vulva (Figs 7, 8, 12, 13). Epigynal plate small, nearly reniform, with swollen posterior margin; copulatory openings hidden in a groove. Spermathecae large and oval; copulatory ducts thin; slender fertilization ducts invisible in dorsal view.

##### Distribution.

China (Zhejiang).

#### Mallinella pluma sp. n.

urn:lsid:zoobank.org:act:0E7F143C-78AA-4CF0-A24D-9F1926101EB8

http://species-id.net/wiki/Mallinella_pluma

Figs 16 –24

##### Type material.

**Holotype**♂,CHINA, Guangxi, Zhuang Autonomous Region: Daming Mountain (23°31'N, 108°21'E; 398m), Wuming County, Nanning City, 20 May 2011, Y. N. Wang leg.

##### Diagnosis.

**Figures 16–21.**
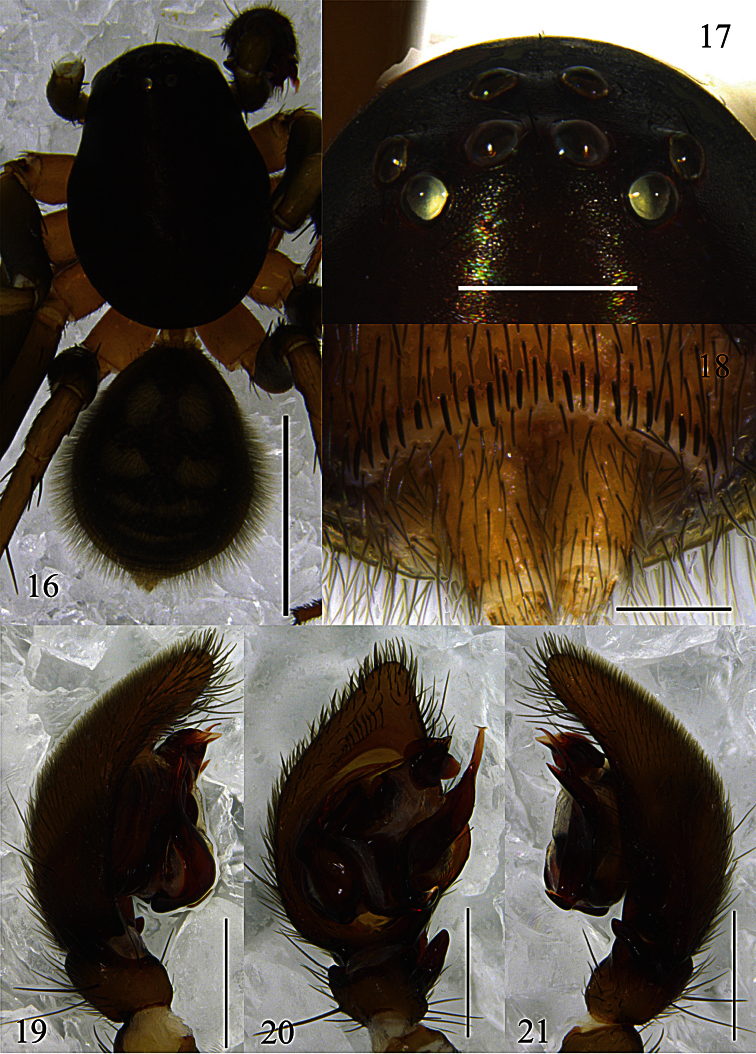
*Mallinella pluma* sp. n., **16** male habitus, dorsal view **17** ocular area, frontal view **18** male, posterior ventral spines, ventral view **19** left male palp, prolateral view **20** same, ventral view **21** same, retrolateral view. Scale bars: 2 mm (**16**); 0.2 mm (**17**); 0.5 mm (**18**–**21**).

**Figures 22–24.**
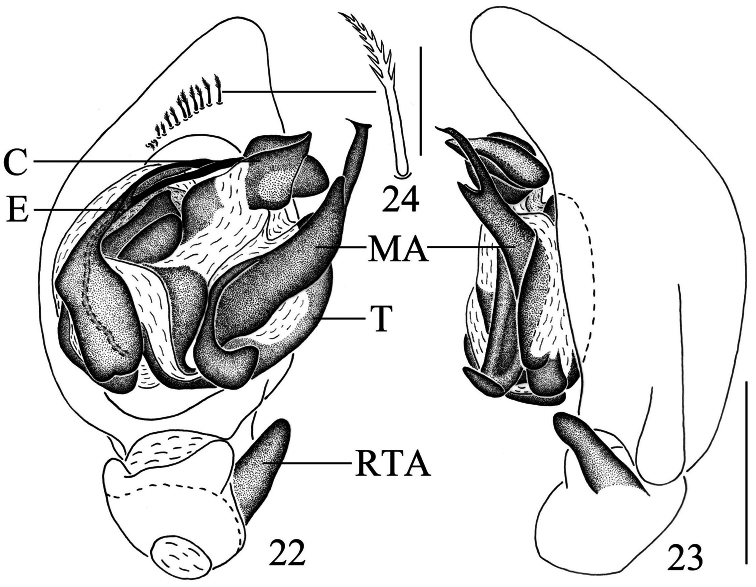
*Mallinella pluma* sp. n., **22** left male palp, ventral view **23** same, retrolateral view **24** a plumose hair (magnified). Scale bars: 0.5 mm (**22–23**); 0.05 mm (**24**).

Male can be easily distinguished from other *Mallinella* males by the extremely narrow and long median apophysis (almost 2/3 cymbium length in ventral view), by the ventral row of plumose hairs near the tip of the cymbium (Figs 20, 22, 24), and also by the dense dorsal hair cover of the opisthosoma (Fig. 16).

##### Etymology.

The specific name is a Latin noun and refers to the plumose hairs near the tip of cymbium.

##### Description.

Male (holotype). Total length 5.30; prosoma 2.86 long, 2.04 wide; opisthosoma 2.24 long, 1.84 wide. Diameters of eyes: AME 0.18, ALE 0.15, PME 0.13, PLE 0.15. Distances between eyes: AME–AME 0.08, AME–ALE 0.10, ALE–ALE 0.50, PME–PME 0.13, PME–PLE 0.23, PLE–PLE 0.73, ALE–PLE 0.05. MOA 0.40 long, front width 0.40, back width 0.38. Clypeal height 0.80. Labium 0.53 long, 0.48 wide. Sternum 1.28 long, 1.22 wide. Measurements of legs: leg I 9.51 (2.30, 0.77, 2.24, 2.35, 1.85), II 8.83 (2.19, 0.82, 1.89, 2.35, 1.58), III 8.57 (2.09, 0.77, 1.73, 2.55, 1.43), IV 11.22 (2.65, 0.82, 2.55, 3.62, 1.58). Leg formula: 4123.

Carapace (Fig. 16) dark brown; median furrow black. Both eye rows (Fig. 17) procurved in dorsal view. Clypeus brown. Chelicerae brown, distally yellowish-brown. Endites yellowish. Labium triangular and brown, distally white. Sternum yellowish-brown and furnished with sparse black setae, lateral margin with small and pointed extensions fitting in coxal concavities of legs. Legs light yellowish-brown except femora that are light brown; metatarsi II–IV distally with ventral hair tufts. Opisthosoma (Fig. 16) oval, longer than wide, dorsally black, with dense hairs and three pairs of white lateral patches (the third slight connected), followed by one transverse white median patch; dorsal scutum indistinct; venter grey-black, covered with three irregular longitudinal white stripes; posterior ventral spines thin (Fig. 18), arranged in a single row. Spinnerets pale yellow.

Palpal organ (Figs 19–24). RTA digitiform, slightly wider at base, gradually tapering towards its blunt apex. Cymbium ventrally with a row of plumose hairs near its tip; cymbial fold broad, more than half as long as cymbium. Apex of median apophysis rostrated and pointing to the prolateral side; the median part of median apophysis with a small digitiform branch; baso-retrolateral fold narrow and long, spatulate, apex blunt. Conductor complex, with a triangular apophysis retrolaterally. Embolus bifurcated at the median part, lateral ramus shorter than mesal ramus, only lateral ramus with subterminal fold.

Female unknown.

##### Remarks.

According to Dankittipakul et al. 2012, this species also should belong to sub-group 3 of the *fronto* species-group, but the plumose hairs on the cymbium ventrally makes that species unique; it is likely that *Mallinella pluma* belongs to an unknown sub-group of the *fronto* species-group.

##### Distribution.

China (Guangxi).

## Supplementary Material

XML Treatment for Mallinella (Mallinella) sphaerica

XML Treatment for Mallinella pluma
